# LSTM-Based GNSS Localization Using Satellite Measurement Features Jointly with Pseudorange Residuals †

**DOI:** 10.3390/s24030833

**Published:** 2024-01-27

**Authors:** Ibrahim Sbeity, Christophe Villien, Benoît Denis, Elena Veronica Belmega

**Affiliations:** 1 Université Grenoble Alpes, CEA-Leti, F-38000 Grenoble, France; 2 ETIS UMR 8051, CY Cergy Paris Université, ENSEA, CNRS, F-95000 Cergy, France; 3 Université Gustave Eiffel, CNRS, LIGM, F-77454 Marne-la-Vallée, France

**Keywords:** satellite selection, single-epoch positioning, machine (deep) learning, long short-term memory neural network, satellite measurement features

## Abstract

In the Global Navigation Satellite System (GNSS) context, the growing number of available satellites has led to many challenges when it comes to choosing the most-accurate pseudorange contributions, given the strong impact of biased measurements on positioning accuracy, particularly in single-epoch scenarios. This work leverages the potential of machine learning in predicting linkwise measurement quality factors and, hence, optimize measurement weighting. For this purpose, we used a customized matrix composed of heterogeneous features such as conditional pseudorange residuals and per-link satellite metrics (e.g., carrier-to-noise-power-density ratio and its empirical statistics, satellite elevation, carrier phase lock time). This matrix is then fed as an input to a long short-term memory (LSTM) deep neural network capable of exploiting the hidden correlations between these features relevant to positioning, leading to the predictions of efficient measurement weights. Our extensive experimental results on real data, obtained from extensive field measurements, demonstrate the high potential of our proposed solution, which is able to outperform traditional measurement weighting and selection strategies from the state-of-the-art. In addition, we included detailed illustrations based on representative sessions to provide a concrete understanding of the significant gains of our approach, particularly in strongly GNSS-challenged operating conditions.

## 1. Introduction

The field of navigation and positioning was revolutionized by the Global Navigation Satellite System (GNSS), which provides precise location services on a global scale. The continual evolution and refinement of GNSS technologies have not only enhanced traditional navigation, but have also opened up new possibilities in diverse fields ranging from transportation and agriculture to surveying and disaster management. To guarantee reliable position estimation, multiple satellite vehicle (SV) selection, fault detection and exclusion (FDE), and weighting techniques must be applied. The challenge is even more important in the single-epoch context, where no access to previous solutions is granted.

Despite these challenges, single-epoch positioning remains essential in several operating contexts. First, it is essential in checking the integrity of the provided solution via the Receiver Autonomous Integrity Monitoring (RAIM) techniques. Second, it can serve as an initial position estimate to initialize the navigation processor [1]. Third, the loosely coupled data fusion of GNSS with an Inertial Navigation System (INS) requires the GNSS outputs (i.e., position and velocity) to be independent. This is to prevent any time-correlated measurements from occurring at the input of the fusion engine. Therefore, single-epoch solutions are necessary. Finally, recently developed Internet of Things (IoT) chips, such as Semtech’s LoRa chip LR1110 [2], utilize low-power receivers to capture a single snapshot of GNSS measurements. These measurements are then transmitted via the IoT network to undergo remote cloud processing. Despite the imposed single-epoch framework, the processing power is not actually limited. In addition, newly developed IoT architectures consider coupling GNSS with low data rate transmissions (e.g., based on LoRa radio technology) for the purposes of remote cloud processing [3].

The GNSS operating conditions, such as Non-Line of Sight (NLOS) and multi-path (MP) propagation, strongly affect the signals received from satellites, resulting most often in strongly biased pseudorange measurements. Accordingly, it is crucial to identify and remove the most-harmful links/satellites that would have a strong contribution to the localization error. With the advancement of GNSS technology, this step has become increasingly challenging with the capability of the receivers to receive tens of satellite signals at each time epoch. The observation noise is related to single-link features such as the signal-to-noise-power-density ratio ($C/N_{0}$) [4] and/or the elevation angle (θ) [5]. Accordingly, widely used pre-selection methods rely on these features for the exclusion or the mitigation of satellites’ measurements that may cause large localization errors [6,7].

To ensure the integrity of the computed solution, several Receiver Autonomous Integrity Monitory (RAIM) and fault detection and exclusion (FDE) techniques have been proposed in the literature, including: RANdom SAmple Consensus (RANSAC) [8], iterative re-weighting [9], subset-testing [10], etc. These techniques entail different trade-offs between computational complexity and performance. Nevertheless, to the best of our knowledge, the satellite selection and weighting challenges remain open to a large extent, taking into consideration the high computational cost of testing all the possible subsets/combinations of available satellites.

Other existing approaches utilize the spatial distribution of positioning solutions conditioned on specific subsets in order to find the satellites that negatively affect the positioning error [11]. The main limitation of such approaches is that they mostly exclude the strongly biased satellites, without mitigating their ranging errors. The exclusion of measurements results in the loss of information, which affects the minimal positioning error that can be achieved. Hence, mitigating measurements’ bias (i.e., through weighting) preserves more information, which helps in providing better performance. In addition, conventional weighting approaches [12] utilize simple parametric functions that depend on linkwise features (e.g., $C/N_{0}$, satellite elevation angle, and other coefficients). Such parametric functions are difficult to fine-tune, which results in sub-optimal positioning errors.

In this paper, to overcome the limitations of conventional satellite selection and weighting algorithms, we propose a unified algorithmic approach that can handle the two problems at once (i.e., satellite selection and weighting) in standalone single-epoch positioning scenarios. One major innovation, hence, resides in the integration of machine learning (ML) in the domain of pseudorange residuals (i.e., joint features), accompanied by other per-link features such as $C/N_{0}$ and the angle elevation, to improve the weights assigned to each satellite in the positioning solver. The preliminary results of this work have been published in [13,14].

ML has a great capability to exploit the hidden correlations in the data, specifically in the extracted features (i.e., the joint and per-link ones) and in the positioning solution. Therefore, we propose a novel ML-based approach based on the potential of an long short-term memory neural network (LSTM NN) fed with a customized feature matrix. This type of neural network (NN) specializes in capturing distant dependencies in its input data, due to its memory cells and its gates (i.e., input, forget, and output gates). In our problem, the LSTM NN is intended to generate predictions of measurement quality factors, which reflect the linkwise standard deviations of pseudorange errors. Subsequently, these predictions are employed to calculate efficient weights for satellites to be further used in a conventional positioning solver such as weighted least squares (WLS).

For training, validating, and testing our NN, we used real data, which were collected during extensive field tests and dedicated measurement campaigns. In total, more than 290 driving sessions were conducted in various operating environments, resulting in 440,000 epochs. The GNSS receiver utilized during these campaigns has the capability of receiving signals from multiple constellations (i.e., GPS, GLONASS, GALILEO, etc.) over multiple bands (e.g., L1, L2, E1, and E5). Also, a high-end GNSS-aided Inertial Navigation System (INS) system was used to collect the ground-truth positions, providing centimeter-level accuracy.

Our contributions in this paper can be summarized as follows. First, we describe a unified innovative solution that simultaneously handles both satellite selection and weighting problems, while exploiting the potential of ML (i.e., LSTM NN). The latter is fed with a customized feature matrix as an input, which includes joint features (i.e., conditional pseudorange residuals) and per-link features (e.g., carrier-to-noise-power-density ratio and its statistics, elevation angle, carrier phase lock time). The rationale behind choosing the LSTM NN is that the customized feature matrix can be viewed as a sequence of pseudo-observations, which can be effectively handled in this type of NN. Second, based on the proposed architecture, we show that more-efficient satellites weights can be computed compared to the conventional parametric state-of-the-art methods based on the performance in terms of positioning error. Third, we validated the proposed algorithmic approach based on real field data, which were collected in a variety of representative environments (including urban, suburban, rural, and even mountainous), hence offering diverse GNSS conditions in terms of propagation and availability. Beyond assessing performance over the entire data set, we also illustrate and analyzed the positioning results achieved on a few typical measurement sessions, hence showing concretely the operating contexts in which the proposed approach is mostly beneficial in comparison with state-of-the-art solutions.

The rest of this article is organized as follows. In Section 2, we introduce the system model, the problem formulation, and existing satellite weighting and selection techniques from the literature. Then, in Section 3, the general architecture of our approach is explained, including the data pre-processing steps and the construction of the input features that are fed into the proposed LSTM NN. Finally, the numerical results obtained on the real collected data are discussed in Section 4.

## 2. Problem Formulation

In this section, we introduce the problem under study followed by a review of the relevant literature, which allows us to position our novel contributions.

### 2.1. Single-Epoch Positioning

For the sake of simplifying the notations and without loss of generality regarding our approach, we first considered a set of *N* single-constellation, single-band pseudorange measurements (Note that we will later broaden the scope of our study to encompass scenarios involving multiple bands and constellations when discussing the experimental results in Section 4.) ${\left\{\rho^{i}\right\}}_{i=1\ldots N}$, where the measurement from the *i*-th SV can be simply modeled as follows:

(1) $$\rho^{i}=\sqrt{{\left(x-x^{i}\right)}^{2}+{\left(y-y^{i}\right)}^{2}+{\left(z-z^{i}\right)}^{2}}+c\;\delta +\eta^{i},$$

assuming the absence of MP or notable biases, where $\rho^{i}$ is the pseudorange between the receiver and the *i*-th SV, with $\left(x^{i},y^{i},z^{i}\right)$ and (x,y,z) the coordinates of the *i*-th satellite and the receiver, respectively. The parameter *c* is the speed of light, and δ is the clock bias between the receiver and the considered constellation. It was assumed that all the necessary corrections, derived from ephemeris data (including SV clock bias, ionospheric and tropospheric delays, Sagnac correction, etc.), have been applied. $\eta^{i}$ is the observation noise, which represents both the receiver noise and other additional errors (e.g., resulting from badly compensated extra delays). Despite the strong correlation of the tropospheric and ionospheric residual errors after the correction from the navigation messages, the latter are usually considered as independent and zero mean in single-epoch processing. Accordingly, the observation noise is assumed to follow a centered Gaussian distribution, i.e., $\eta^{i}∼N\left(0,\;{\left(\sigma^{2}\right)}^{i}\right)$.

Our aim is to estimate the vector $X={\left[x,y,z,\delta \right]}^{⊤}$ from the set of measurements ${\left\{\rho^{i}\right\}}_{i=1\ldots N}$. An efficient solution that is widely used is the maximum likelihood estimator (MLE) [15], which simplifies to a WLS estimator for our Gaussian noise model:

(2) $$\hat{X}=\arg\;\min_{X}\;\sum_{i=1}^{N}\omega^{i}(\rho^{i}-h^{i}\left(X\right){)}^{2},$$

where the observation function for the *i*-th satellite is defined as

(3) $$h^{i}\left(X\right)=\sqrt{{\left(x-x^{i}\right)}^{2}+{\left(y-y^{i}\right)}^{2}+{\left(z-z^{i}\right)}^{2}}+c\;\delta ,$$

and the optimal weights are equal to

(4) $$\omega^{i}=\frac{1}{{\left(\sigma^{2}\right)}^{i}}.$$

The solution can be computed using an optimization algorithm such as Levenberg–Marquardt or Gauss–Newton [16].

### 2.2. GNSS Satellite Selection and Weighting Problems

In general, the exclusion of presumably strongly biased measurements is performed using an initial rough rejection step based on thresholding over the $C/N_{0}$ or satellite elevation angle values. This parametric rejection is followed by a weighting step using empirical functions that estimate the per-link standard deviation of the accepted measurements [12], given as follows:

(5) $${\left(\nu^{2}\right)}^{i}=\frac{1}{\sin^{2}\left(\theta^{i}\right)}\left(\sigma_{\rho Z}^{2}+\frac{\sigma_{\rho c}^{2}}{{\left(C/N_{0}\right)}^{i}}+\sigma_{\rho a}^{2}{\left(a^{2}\right)}^{i}\right).$$

This function mainly depends on $C/N_{0}$, the elevation angle θ, and the range acceleration *a*, in addition to other coefficients ($\sigma_{\rho Z}^{2},\sigma_{\rho c}^{2},\sigma_{\rho a}^{2}$), which are challenging to fine-tune and cannot easily be generalized.

The accuracy of the resulting estimated position may be significantly degraded because of the strongly biased measurements that are induced by MP, hence violating the underlying Gaussian-centered model. Hence, the exclusion or de-weighting (i.e., assigning zero weights) of such problematic measurements represents a key processing step. Innovation monitoring tests [17] at the navigation processor stage are capable of efficiently detecting this type of biased measurement. However, the convergence of the tracking filter is required, in addition to an accurate predicted state: including position, receiver clock offset, etc.

In the context of single-epoch processing, there is no predicted solution available, and the selection of SVs depends solely on the measurements, with minimal prior information. This means that detecting *k* faults among *N* measurements leads to a combinatorial number of subsets ($C_{k}^{N}$) to be tested in the case of an exhaustive search, making it prohibitive for real-time applications. To illustrate this precisely, consider a representative scenario with a maximum of 10 faults among 40 measurements; in this case, more than $847\times 10^{6}$ subsets have to be tested, which is computationally unfeasible.

### 2.3. Existing Works

As we have already mentioned, detecting and excluding strongly biased or corrupted signals comprise an important challenge in GNSS to improve its integrity. For this purpose, various RAIM techniques have been proposed in the literature. They include classical FDE [18], brute force subset testing [19], Advanced Receiver Autonomous Integrity Monitoring (ARAIM) [18,20], and range consensus (RANCO) [11].

The classical FDE [18,19,20,21] employs a dual-statistical assessment of measurement residuals through global and local tests. In the global test, the residuals are assumed to follow a centered normal distribution. Accordingly, it follows that the weighted squared sum of the residuals should conform to a chi-squared distribution. The empirical statistic is then compared to this expected chi-squared distribution, while specifying a false alarm probability. If a fault is detected in the global test, the local test is used to detect the faulty measurement and exclude it. This is performed by excluding the measurement that has the highest residuals. Measurements are then excluded sequentially in a loop until the global test stops detecting a fault. Although this approach is efficient in excluding faulty measurements, it utilizes the least squares (LS), which is very sensitive to outliers. This makes its loop less efficient, resulting in the wrong exclusions.

The brute force subset testing approach [18,22] assumes the existence of *k* faulty measurements among the *N* received measurements. All the possible subsets constituted of *k* out of *N* measurements are constructed, and their residuals are tested using the global test. Based on the latter, the subset with the best score is selected. The main disadvantage of this approach is its computational cost, which is generally not tractable, especially in real-time systems.

To reduce the computational cost, other approaches have been proposed, such as range consensus (RANCO) [23]. This approach hinges on the initial selection of a subset of measurements, determined by a specific condition (e.g., Geometric Dilution of Precision (GDOP)), and subsequently, deriving a solution through computation. Then, all the measurements are compared to this solution based on a selected threshold, while the pseudorange residuals are categorized into inliers (for values below the threshold) and outliers (for values above the threshold). Subsequently, the inliers from the subset that receives the highest consensus are selected, while the outliers are rejected. Iteratively and conditionally, additional subsets can be considered and compared.

At last, other approaches perform statistical tests in the solution domain (by utilizing the solution’s covariance matrix), rather than the residuals space. As an example, consider the Multi-Hypothesis Solution Separation (MHSS), which is part of ARAIM [24,25]. This approach calculates a preliminary position from all the received measurements and compares it with the solutions estimated from all the possible subsets of measurements. Upon the detection of a fault using local tests, an exclusion attempt of the faulty measurement is performed using the subset approach. The main disadvantage of this approach resides again in its computational cost.

All the above approaches are standalone and do not have access to previous position estimates. A more-recent solution proposed in [26] relies on a new FDE, which uses a standalone FDE block along with an FDE-based Extended Kalman Filter (EKF). The standalone part of this approach is based on a residual test using WLS. The utilization of each system part depends on both the covariance matrix and a predefined threshold. More precisely, when the covariance matrix falls below a certain a priori threshold (elementwise), the FDE-based EKF is used; otherwise, a standalone FDE is applied. This algorithm is based on a subset testing method for SV selection and a standard WLS for position computation.

In comparison with the previous existing approaches, the method in [26] was shown to significantly improve the positioning performance. This approach was compared with both the well-known GNSS software RTKlib 2.4.2 and the solution delivered by the Ublox receiver. Furthermore, it was evaluated in single-epoch mode and filtered mode. The first comparison was performed in single-epoch mode with respect to the RTKlib solution. Multiple tests were performed over different sessions, where this approach showed a good performance over the RTKlib solution, as shown in Table 1.

The second comparison was performed in filtered mode with respect to the solution provided by the Ublox chip, since only the Ublox chip provides a filtered solution. Different Ublox chips were used (i.e., NEO-M8P and ZED-F9P), both using the Airborn 4G filtering profile. The comparison was performed in various operating conditions such as urban, suburban, highways, etc. As shown in Table 2 and Table 3, the solution provided by the approach in [26] yielded a better performance with respect to the solution provided by the Ublox chip in filtered mode.

Due to the good performance demonstrated by the approach in [26] compared to two representative and well-known approaches, we chose it as a reference benchmark in this paper. As our study is focused on single-epoch localization, we considered only its standalone part.

As opposed to the aforementioned state-of-the-art, our work is the first to propose a unified ML-based satellite selection and weighting approach using an LSTM NN. The satellite selection problem is cast into a weighting problem, where nearly zero weights are assigned by our algorithm for satellite measurements that should be excluded. Another novelty of our approach lies in exploiting per-link features (i.e., $C/N_{0}$, elevation angle, etc.) jointly with joint features (i.e., residual matrix) to efficiently weight satellite measurements.

## 3. Proposed System Architecture

Our main goal is to efficiently weight the contributions of all the satellite measurements while estimating a position, which requires exploiting the complex hidden inter-dependencies across multiple radio links from distinct satellites. For this purpose, we used deep learning (DL), a powerful toolbox, which has the ability to exploit such complex hidden inter-dependencies.

**Figure 1:**
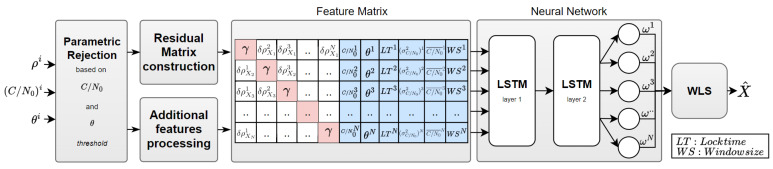
Complete architecture of our proposed approach.

We start by constructing a comprehensive and representative feature set to be provided as an input to the chosen NN. A distinctive aspect of our approach is that we incorporated joint and per-link features in a unique comprehensive input matrix. The construction of this matrix will be explained in detail in the following sections. This matrix will be fed to the NN (i.e., LSTM NN), which will be trained to predict the weights ${\hat{\omega }}^{i}$, which are hopefully related to the underlying distribution of pseudorange errors according to (4). Our intuition and expectation are that the strongly biased pseudorange measurements will be excluded by the NN by assigning them close-to-zero weights. In summary, the satellite selection problem is turned into a soft weighting problem. The complete proposed architecture is shown in Figure 1.

### 3.1. Data Normalization and Parametric Rejection of Outliers

When it comes to deep-learning-based approaches, aside from choosing the relevant input features discussed in the next subsections, the value ranges of the input features are also crucial. This is especially true for LSTM NNs, which do not handle large values well. To tackle this issue, we can normalize the input features. However, the challenge behind huge data outliers (i.e., unusual data points) still remains. The presence of such outliers has a negative impact on the normalization scale, may suppress important information, and should be rooted out from the start, as shown in Figure 1. Indeed, our analysis of the collected data showed that the worst outliers, caused by some corrupted signals, led to significant errors in the pseudorange residuals, which are the main components of our proposed joint features (see Section 3.3).

**Figure 2:**
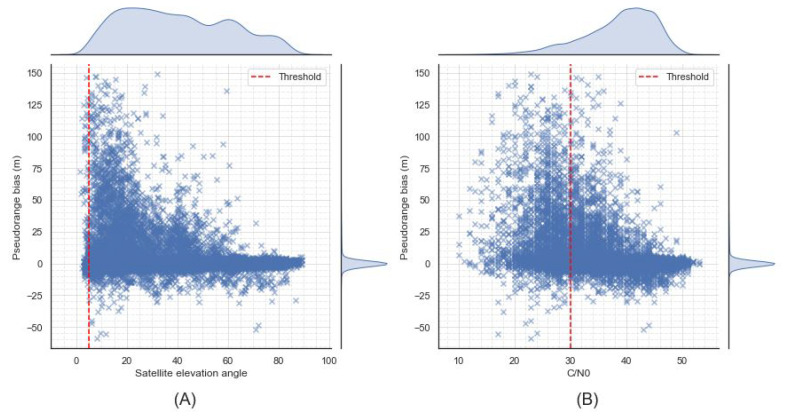
Data analysis for parametric rejection of outliers: (**A**) pseudorange bias (m) vs. satellite elevation angle and (**B**) pseudorange bias (m) vs. $C/N_{0}$.

In order to visualize the outliers, in Figure 2, we show scatter plots of the pseudorange bias versus the elevation angle (Figure 2A) and the pseudorange bias versus $C/N_{0}$ (Figure 2B), calculated from the ground-truth position data. It can be observed that low elevation angles and low values of $C/N_{0}$ will increase the probability of having a biased pseudorange, as expected. Although the proportion of such corrupted signals is relatively small, as can be seen from the shaded histograms in Figure 2A,B, as mentioned above, it is important to exclude them using parametric rejection via thresholding, prior to weighting.

On the other side, a too-severe parametric rejection incurs the risk of losing informative satellite signals that would be useful to our ML model. Thus, a careful balance between the negative outliers’ effect and losing informative signals has to be reached. Our exhaustive tests showed empirically that the rejection threshold values that reach a good balance and lead to the best positioning performance are: 30 dB-Hz for the $C/N_{0}$ and $5^{\circ }$ for the elevation angle.

### 3.2. Per-Link Features

Numerous per-link measurement features may contain valuable information with respect to positioning and, hence, accordingly, may be beneficial to the NN as inputs. These include the SV elevation angle θ, the carrier phase lock time, $C/N_{0}$, and its empirical statistics.

For instance, as shown in Figure 2A, SV signals with low elevation angles have a higher probability of being strongly biased. This is mainly caused by the fact that the signals received under such angles tend to travel longer distances in the ionospheric and tropospheric layers of the atmosphere. Likewise, they are more prone to suffer from severe NLOS conditions. Besides, the carrier phase lock time somehow indicates if a signal is newly acquired.

In Figure 2B, one can also note that SV signals with low $C/N_{0}$ have a higher probability of being strongly biased. Intuitively, these signals have a higher tracking noise in the ranging processor, which also leads to a higher pseudorange measurement noise.

Beyond this, as pointed out in [27], the empirical statistics of $C/N_{0}$ (i.e., its variance $\sigma_{C/N_{0}}^{2}$ and mean $\bar{{\left(C/N_{0}\right)}^{i}}$, calculated over a short period of time) can also reflect the current MP operating conditions. While our work focuses on single-epoch processing, we can still effectively estimate the variance of $C/N_{0}$ over short intervals of a (past) few seconds. This approximation will primarily impact measurement weights and introduce negligible correlation among them.

**Figure 3:**
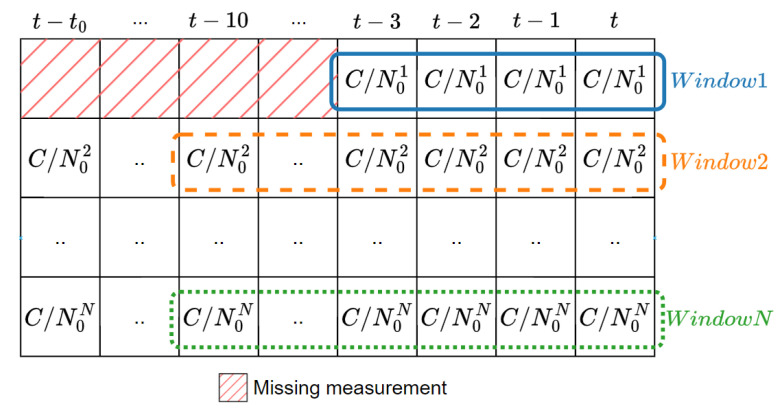
Variable size sliding window for $C/N_{0}$ variance and mean calculation.

As a new SV can be acquired or lost during a navigation session, the number of consecutive measurements of $C/N_{0}$ may change. Hence, the sliding window can vary in size (as illustrated in Figure 3), with a maximum of 10 epochs (which is equivalent to 2 s). In the case of a newly acquired SV, where only one entry is present in the window, an arbitrary large variance value is assigned. Thus, we also make use of this window size as an extra feature. On its own, it indeed indicates if the quality of the received signal is erratic in the short-term and/or if the amount of related information is too limited.

To sum up, the above acquired per-link features are combined into a sub-matrix that comprises six feature column vectors, each with a dimension *N* (see Figure 1).

### 3.3. Joint Features

Joint features accounting for the simultaneous impact of multiple measurements over distinct links (i.e., in contrast to per-link features) can also be extracted from the comparison of the positioning solutions from different tested subsets. To overcome the challenge of testing all the possible subset combinations, which is computationally prohibitive, our approach leverages the ability of ML to extract hidden inter-dependencies from only *N* such subsets.

At each navigation epoch, we assume that multiple (i.e., *N*) satellite signals are received, and a new matrix of positioning residuals M is constructed, as follows. We generate *N* subsets $S_{n}$ of N−1 satellites, where we exclude one distinct satellite (i.e, the *n*-th satellite) at a time.

(6) $$S_{n}=\left\{\rho^{i}\right\},\;i=1\ldots N,\;i\neq n$$

For each subset $S_{n}$, we calculate the corresponding solution $X_{n}$ using (2) with equal weights. Then, for each of the *N* resulting positions $\left\{X_{1},...,X_{N}\right\}$, we calculate the N−1 pseudorange residuals:

(7) $$\delta \rho_{X_{n}}^{i}=\rho^{i}-h^{i}\left(X_{n}\right),\;\;i\neq n$$

The coefficient ${\left[M\right]}_{n,i}$ (i.e., row *n*, column *i*) of the residual matrix M is then simply given by the corresponding residual for non-diagonal coefficients or by an arbitrary large value γ for the diagonal terms, indicating that the satellite has been deliberately excluded.

(8) $${\left[M\right]}_{n,i}=\left\{\begin{array}{c} \delta \rho_{X_{n}}^{i},\;\;i\neq n\\ \gamma ,\;i=n \end{array}\right.$$

Each row *n* of the matrix will, thus, provide residuals associated with the exclusion of the *n*-th measurement. Although it assumes a single fault per subset (i.e., row), our intuition is that such a matrix is able to reveal the complex joint contributions of each satellite to the positioning solution, while being fed as a single input to the NN.

### 3.4. Long Short-Term Memory Neural Network

Obtaining the best performance of the NN requires choosing a suitable architecture for the investigated problem. This may be a challenging task, taking into consideration the wide variety of possible NNs. For this purpose, an empirical evaluation of different types of NNs was performed [14]. The tested NN architectures included convolutional neural networks (CNNs), fully connected neural networks (FCNNs), and various other types of recurrent neural networks (Simple-RNN, LSTM [28], Bi-LSTM [29], and gated recurrent units (GRUs)). Among the tested architectures, the best positioning performance, taking into consideration the complexity of the NN, was obtained by the LSTM NN. Accordingly, this architecture was selected and further optimized as explained in Section 4.2.1.

The overall input matrix of features being fed to the NN can be seen as a sequence of *N* pseudo-observations. At each observation, a single pseudorange measurement is excluded from computing the solution. By analyzing this sequence, the LSTM NN can exploit the correlations between the excluded measurement and the solution and pinpoint which measurement exclusions have the best impact on the quality of the positioning solution. As a result, the NN will be capable of predicting weights that exclude multiple biased measurements by analyzing the sequence of pseudo-observations, set as joint features (see Section 3.3). Besides, the additional per-link features for the excluded measurements were concatenated for each pseudo-observation to provide more information about the excluded satellites (see Section 3.2).

In this kind of problem, the LSTM NN architecture, which is a type of recurrent neural network (RNN) [30], has the advantage of keeping memory over multiple (possibly distant) pseudo time steps. Hence, it is also suited to exploiting the correlations across the matrix rows in our case, even if we explicitly deal with a single-epoch problem. Similar applications of the LSTM NN to other time-invariant problems have already been considered. For instance, in [31], an LSTM NN was used to process data with long-range interdependence (i.e., using the geometric properties of the trajectory for unconstrained handwriting recognition).

Note that several other (more-complex) NN architectures were evaluated. For instance, we considered a more-complex architecture composed of two different concatenated NNs. The first NN processes only the residual matrix as the input. Its output is concatenated with the additional per-link features and fed as the inputs to the second fully connected NN (FCNN). However, such architectures did not provide any significant improvement over the LSTM NN, which only processes our constructed feature matrix.

## 4. Numerical Results

To assess the feasibility of our approach, the selected neural network architecture was first optimized. Then, a comprehensive performance assessment was performed based on the collected real-world measurement data, as described below.

### 4.1. Data Collection and Scenarios

To train, validate, and test our approach, we exploited real field data, which were collected through extensive measurement campaigns. These campaigns were conducted in various operating conditions and environments in different cities and countries (e.g., France and the USA), including dense urban areas, open skies, and different mobility scenarios. A multi-sensor platform developed by CEA-Leti, named *Vehloc*, was used for the data collection. This platform offers the possibility to integrate two different receiver chips: either a NEO-M8P or a ZED-F9P, both manufactured by Ublox. The ZED-F9P, a dual-band RTK GNSS receiver, has the capability of receiving up to N=184 satellite signals from multiple constellations (e.g., GLONASS, GALILEO, GPS, etc.) at different frequencies (E1, E5, L1, and L2 bands). The NEO-M8P chip has the capability of receiving signals from multiple constellations (only GLONASS and GPS were used), but it operates on single-band L1 signals. The raw measurements, including: the pseudorange, SV ID, frequency ID, constellation ID, $C/N_{0}$, pseudorange rate carrier phase, carrier phase lock-time, etc., were collected at a 5 Hz rate, along with the broadcast ephemeris.

**Figure 4:**
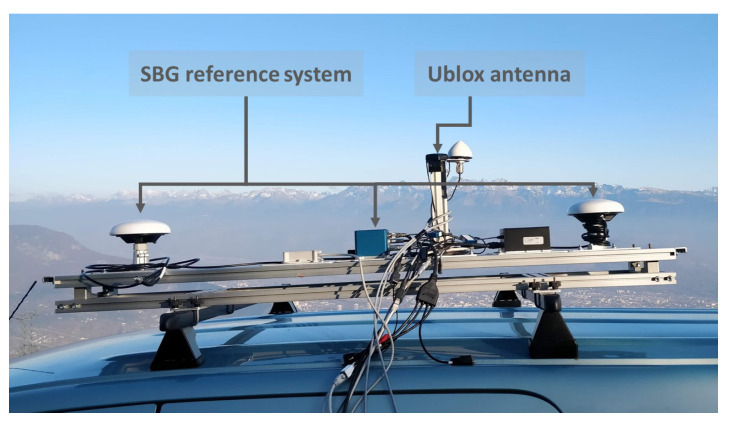
Experimental setup used for measurement data collection.

The raw reference position was first acquired by a high-end, GNSS-aided INS Ekinox platform from SBG. These collected data were then post-processed using the Qinertia 3.4.304 software, also from SBG, to obtain the final reference positions within centimeter-level accuracy, even in the most-challenging operating conditions. The experimental setup, which was installed on the rooftop of a vehicle, is shown in Figure 4. The picture shows two essential components of the experimental setup, namely the SBG reference system and the Ublox antenna of the Vehloc platform.

Overall, a total of 291 sessions, comprising 440,000 epochs, were carried out during the data collection, resulting in a comprehensive, diverse, and extensive dataset, which we used to validate our approach.

### 4.2. Evaluation Results

**Figure 5:**
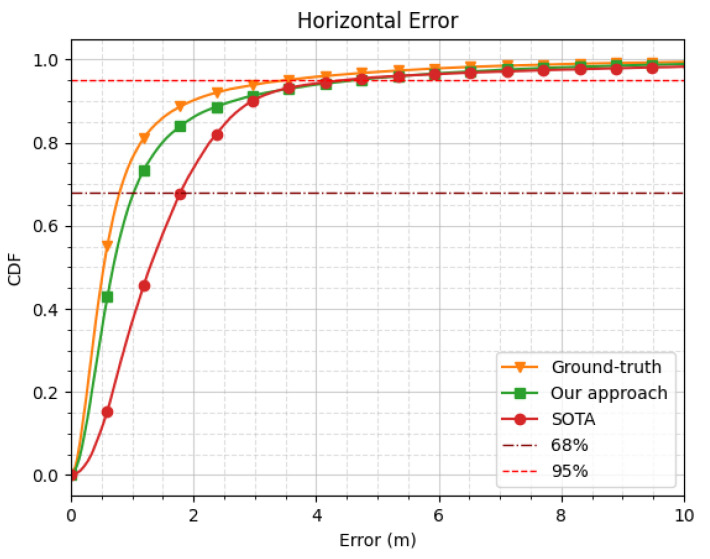
Empirical cumulative density function (CDF) of horizontal error for various measurement weighting/selection strategies on real field data (over the unseen test dataset of the epochs from the ZED-F9P receiver).

**Figure 6:**
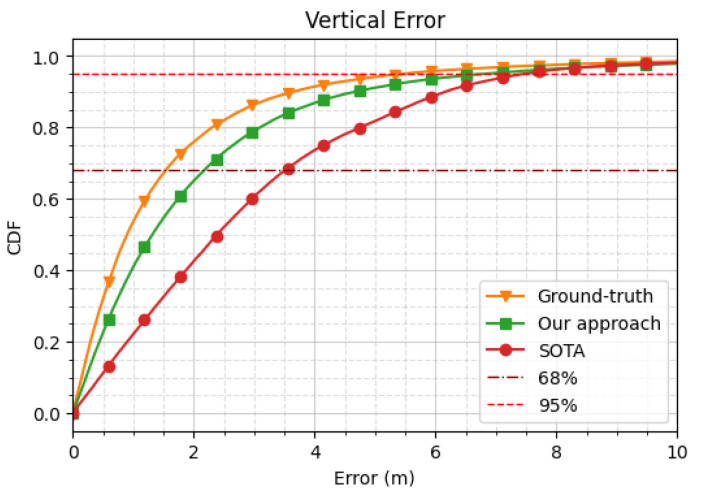
Empirical CDF of vertical error for various measurement weighting/selection strategies on real field data (over the unseen test dataset of the epochs from the ZED-F9P receiver).

As we are following a supervised training for our NN, an important aspect is the ground-truth labels that are used for training. Ideally, the labels shall be the optimal weights. However, optimal weights in our case are not well-defined for several reasons. From the Bayesian estimation perspective, this would require having access to the true distribution (i.e., the standard deviation in particular, assuming a Gaussian distribution of the measurements errors), while only a single sample of this distribution is available. Indeed, having more-precise information about the underlying distribution would require collecting multiple measurements from the same receiver’s position, also with the same SV positions, which is not feasible. When dealing with a single sample/observation, the most-accurate estimate of the standard deviation is the absolute value of the error calculated specifically for that measurement. This approach is further reinforced by empirical evidence, by utilizing the weights as:

(9) $$\begin{array}{c} \omega^{i}=1/{\left(\rho^{i}-h\left(X_{true}\right)\right)}^{2} \end{array},$$

where $X_{true}$ stands for the ground-truth position collected from the reference system. Utilizing this weighting method has provided very good results, as shown in Figure 5 and Figure 6; thus, these weights were used as the data labels.

In the following sections, we will compare the performance of three different approaches from various perspectives. The first approach, referred to as the “ground-truth”, utilizes weights as in (9) with the ground-truth position, before applying WLS positioning. The second approach, named “our approach”, exploits the overall feature matrix comprising the two sub-matrices (i.e., the “residual matrix” concatenated with the additional per-link features) as an input to the NN to predict linkwise quality factors, which are then used to compute the weights as in (4) for WLS positioning. Finally, in the third “SOTA” method, the state-of-the-art solution from [26] (see Section 2.3) is used for positioning.

#### 4.2.1. Neural Network Optimization

To improve the performance of our NN architecture as much as possible, we focused on choosing the best possibilities (out of the tested possibilities) for two primary hyperparameters: the number of hidden layers and the number of neurons per layer. In addition, our consideration of the training time of the NN led us to the selection of other relevant hyperparameters, such as the activation function for each layer. Specifically, given the strictly positive nature of the label weights, the rectified linear unit (ReLU) activation function was employed for the output layer. Also, the Adam optimizer was used to train the NN due to its computational efficiency [32].

To mitigate the computational complexity associated with an exhaustive search over all possible combinations of the number of neurons and the number of hidden layers, we adopted an incremental approach. The number of neurons was systematically increased based on a heuristic rule of thumb commonly used in machine learning, where the number of neurons in a hidden layer is a multiple of the number of input features.

We proceeded in two steps to evaluate the performance of the tested NN architectures and prevent overfitting. Firstly, the dataset was partitioned into three disjoint subsets, allocating 60% for training, 20% for validation, and 20% for testing. Secondly, an early stopping callback mechanism, utilizing the validation subset, was implemented to terminate the training process in the absence of further performance improvement.

After the training and validation steps, the performance evaluation was performed on unseen data (i.e., test subset) to measure the effectiveness of the model in an unseen context. This overall process in training, testing, and optimizing the architecture of the NN contributes to creating a robust and reliable model.

**Figure 7:**
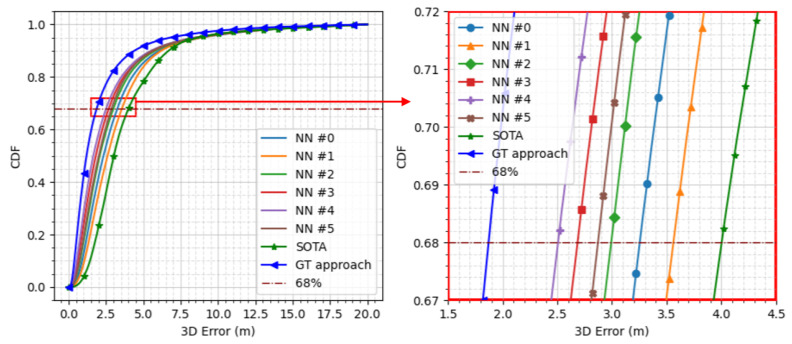
Comparison of the cumulative density function of different NN architectures on unseen (test) data.

The CDF of the 3D positioning error for a small sample of the tested NN architectures is shown in Figure 7. The description of each of the labeled architectures is as follows:

- NN #0: an LSTM NN with 1 hidden layer of 550 neurons.
- NN #1: an LSTM NN with 2 hidden layers: 330 neurons in the first hidden layer and 220 neurons in the second hidden layer.
- NN #2: an LSTM NN with 2 hidden layers: 550 neurons in the first hidden layer and 440 neurons in the second hidden layer.
- NN #3: an LSTM NN with 1 hidden layer of 1100 neurons.
- NN #4: an LSTM NN with 2 hidden layers: 990 neurons in the first hidden layer and 880 neurons in the second hidden layer.
- NN #5: an LSTM NN with 3 hidden layers: 110 neurons in the first hidden layer, 440 neurons in the second hidden layer, and 550 neurons in the third hidden layer.

All the compared NNs had better performance with respect to the SOTA approach. However, the best performance was from NN #4, which achieved 2.5 m of 3D positioning error at 68% (i.e., 37.1% improvement compared to the SOTA approach.)

#### 4.2.2. Performance on Representative Sessions

**Figure 8:**
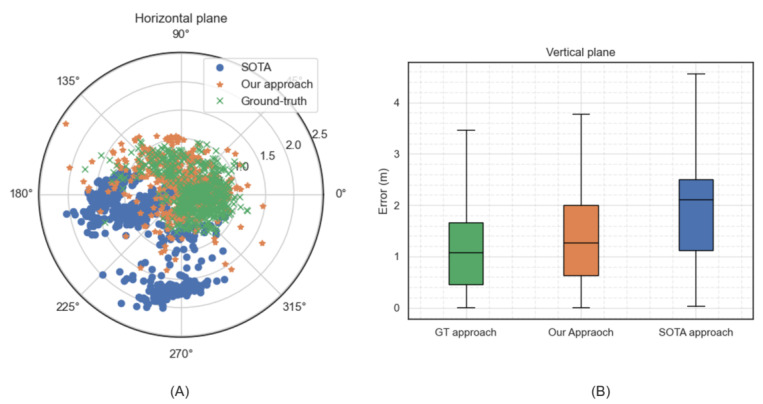
The positioning error traces in the horizontal plane as shown in (**A)** and in the vertical plane as shown in (**B**) for our proposed approach (in orange color), the state-of-the-art approach (in blue color), and the ground-truth approach (in green color) for Session 1.

**Figure 9:**
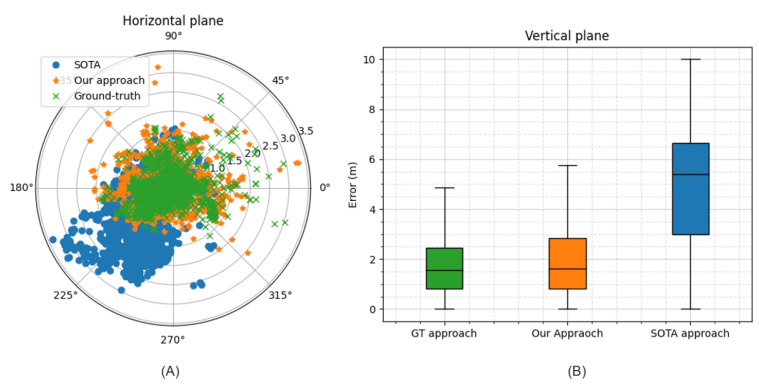
The positioning error traces in the horizontal plane as shown in (**A**) and in the vertical plane as shown in (**B**) for our proposed approach (in orange color), the state-of-the-art approach (in blue color), and the ground-truth approach (in green color) for Session 2.

In order to gain a better understanding and visualize the difference in performance between the state-of-the-art (SOTA) and our approach, we examined two vehicular sessions: Session 1 was an open-sky session, where we illustrate our typical relative improvement with respect to the SOTA approach in typical operating conditions. The horizontal and vertical errors for this session are represented in Figure 8A and Figure 8B, respectively. Session 2 was a different session in a more-GNSS-challenged environment, where a significant part of this session was under solar panels. The latter caused a significant obstruction of the signals, in addition to MP propagation conditions. The horizontal and vertical errors for this session are represented in Figure 9A and Figure 9B, respectively.

In Session 1, the average 3D positioning error for the SOTA approach was 2.37 m and 1.52 me for our approach. As can be seen from Figure 8A, most of the horizontal errors for our approach fell within the radius of 1 m, while for the SOTA approach, most of the errors fell within the radius of 1.75 m. Besides, the scattering of the horizontal errors of the SOTA approach on a wider radius compared to ours highlights the robustness of our approach. Figure 8B shows the superiority of our approach also in terms of the vertical error: the interquartile range of our approach was indeed much closer to the ground-truth than the SOTA approach.

In Session 2, the superiority of our approach was even more evident. In this session, the average 3D positioning error for the SOTA approach was 5.19 m and 2.16 m for our approach. The SOTA approach’s performance was lower compared to Session 1 because it failed to reject at least one strongly biased measurement, which was apparently present for most of the session duration (likely due to similar multipath propagation conditions) and affected the overall performance. This can be seen clearly in Figure 9A, where the horizontal positioning errors of the SOTA approach are systematically shifted in the same direction. On the contrary, our approach was capable of excluding such strongly biased measurements. Furthermore, the interquartile ranges of the vertical errors in Figure 9B again show the superiority of our approach in terms of the vertical error as well. Finally, it is worth highlighting that our approach exhibited a greater magnitude of improvement in increasingly GNSS-challenged condition, as interpreted in these two sessions.

As previously mentioned, our approach was designed to address both the satellite-measurement-selection problem and the weighting problem. To assess and illustrate its effectiveness across these dimensions, we chose two more distinct navigation sessions conducted in very different operating environments: one in a country-side setting and another in an urban area.

The rationale behind selecting these specific operating conditions is as follows. In country-side areas, during navigation sessions, the likelihood of encountering signals in NLOS conditions is relatively low. In this session, the average SV acceptance rate in the SOTA algorithm was 73.5% per epoch. Consequently, the primary challenge lies in effectively weighting the satellite measurements. Conversely, in urban areas, there is a higher probability of receiving strongly biased measurements due to NLOS conditions. In this session, the average SV acceptance rate in the SOTA algorithm was 53.1% per epoch. This poses the challenge of excluding the strongly biased measurements.

**Figure 10:**
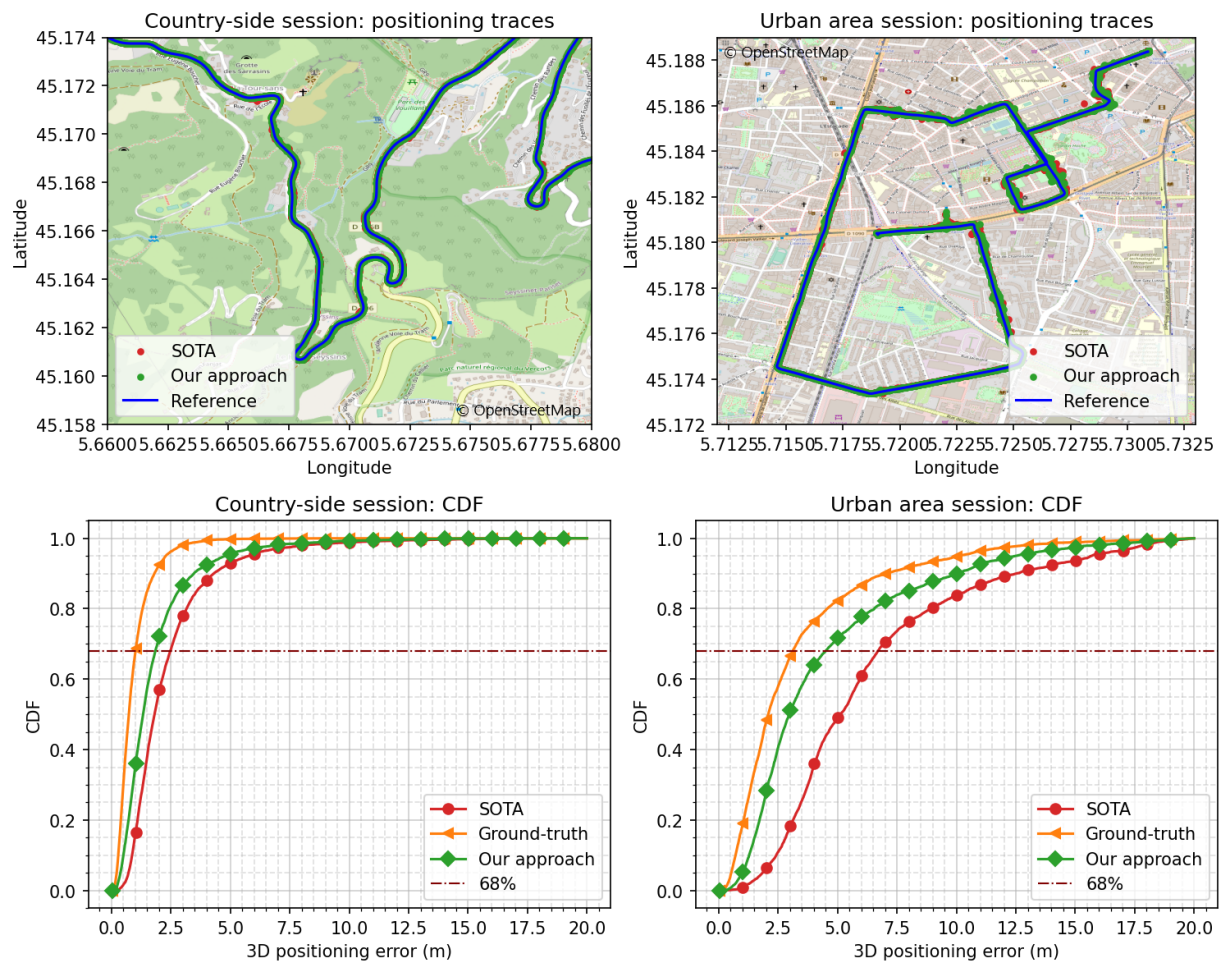
Performance comparison of the SOTA, the ground-truth, and our proposed approach in a country-side and an urban area session.

In Figure 10, we present two sessions from both operating conditions alongside their CDF of the 3D positioning error for three approaches: the SOTA approach, our proposed approach, and the ground-truth approach. In both operating environments, our approach yielded an improved performance compared to the SOTA approach. In particular, in the country-side session, we obtained a 25.2% (at a 68% CDF) improvement, while for the urban area session, we achieved a 32.9% improvement compared to the SOTA.

#### 4.2.3. Overall Performance

Finally, Figure 5 and Figure 6 show the empirical CDF of the horizontal and vertical positioning errors obtained with the three different weighting strategies (i.e., the ground-truth, the SOTA, and our approach) over the unseen testing subset from all the navigation sessions from the ZED-F9P receiver (77,200 epochs).

In these figures, we observe a significant improvement in terms of both the horizontal and vertical errors of the our approach compared to the state-of-the-art solution. Indeed, our approach exhibited a performance gain with respect to the SOTA approach of 0.76 m (i.e., 42.7%) in terms of the horizontal error at a 68% CDF. As for the vertical error, we also observed an improvement of 1.34 m (i.e., 38.1%) at a 68%CDF.

At last, note that a similar performance was also obtained when tested on the NEO-M8P data. To avoid redundancy, we do not include the corresponding figures in this paper.

## 5. Conclusions

In this paper, we introduced a novel approach for SV measurement weighting in single-epoch GNSS positioning by exploiting a combination of joint features (i.e., conditional pseudorange residuals) and per-link features (i.e., $C/N_{0}$ and its empirical statistics, satellite vehicle elevation angle, carrier phase lock time). Our approach relies on an LSTM neural network in predicting several quality factors to weight the contributions of different measurement into the calculation of a position. Our results on real data obtained from field experimental sessions demonstrated the robust performance of the proposed solution in challenging environments, while outperforming a recent state-of-the-art approach. Furthermore, to provide a more-concrete understanding of the significant gains and benefits observed with our approach, we included detailed numerical results obtained in representative sessions. To sum up, our proposed solution is particularly promising in IoT applications (remote processing), where accurate single-epoch positioning is essential, whether in real-time or offline.

The main contributions of our work can be summarized as follows:

(i) A unified ML-based approach was proposed for satellite selection and weighting, using heterogeneous input features.
(ii) Our approach was validated using real data. These data were collected from extensive data-collection campaigns, which included diverse operating conditions (i.e., urban, suburban, countryside, etc.).
(iii) Our approach exhibited significant improvement with respect to recent SOTA approaches of 42.7% (respectively, 38.1%) at a 68% CDF, in terms of horizontal (respectively, vertical) error.

## Figures and Tables

**Table 1:** Performance comparison in terms of horizontal and vertical errors in single-epoch mode: RTKlib versus the approach in [26].

	Horizontal Error (m)			Vertical Error (m)		
	**RMS**	**P68**	**P99**	**RMS**	**P68**	**P99**
RTK lib	1.45	1.09	4.47	2.36	1.32	8.68
[26] approach	0.63	0.61	1.45	1.08	1.08	2.42

**Table 2:** Performance comparison in terms of horizontal and vertical errors in filtered mode: the Ublox solution (NEO-M8P) versus the approach in [26].

	Solution	Normal		Degraded	
		**P68**	**P99**	**P68**	**P99**
Horizontal Error (m)	Ublox filtered (Airborn 4G)	2.09	4.62	2.69	6.92
	[26] filtered	1.7	3.97	2.13	6.95
Vertical Error (m)	Ublox filtered (Airborn 4G)	3.21	6.28	3.93	9.48
	[26] filtered	3.17	5.11	3.33	6.6

**Table 3:** Performance comparison in terms of horizontal and vertical errors in filtered mode: Ublox (ZED-F9P) versus the approach in [26].

	Solution	Normal		Degraded	
		**P68**	**P99**	**P68**	**P99**
Horizontal Error (m)	Ublox filtered (Airborn 4G)	1.84	4.94	2.97	5.83
	[26] filtered	1.48	3.6	2.25	4.10
Vertical Error (m)	Ublox filtered (Airborn 4G)	3.00	9.64	6.97	12.02
	[26] filtered	2.85	5.25	3.62	5.81

## Data Availability

The datasets used in this article are not readily available for intellectual property reasons.
